# Being and doing anorexia nervosa: An autoethnography of diagnostic identity and performance of illness

**DOI:** 10.1177/13634593211017190

**Published:** 2021-05-27

**Authors:** Lauren O’Connell

**Affiliations:** Sociology department, Colchester campus, UK

**Keywords:** anorexia nervosa, autoethnography, diagnostic identity, eating disorder treatment, iatrogenesis

## Abstract

This autoethnography examines my experience of the diagnosis and treatment of anorexia nervosa. Drawing on memory and personal and medical documents relating to inpatient admissions in an adult specialist eating disorder unit, I narrate and analyse my experience in terms of my relationship to the diagnosis of anorexia and the constructions of it I encountered. I show how I came to value an identity based on anorexia and how I learned ways of ‘doing’ the diagnosis in treatment. This involved me valuing medical markers of illness, including signs of poor health, which became crucial to how I performed my diagnosis and retained the diagnostically-informed sense of self that I valued. I suggest that, ultimately, these diagnostic-dynamics, alongside other effects of long-term inpatient treatment such as detachment from ‘normal life’, prolonged my struggles with self-starvation. The insights from this autoethnography shed light on potential iatrogenic impacts of diagnosis and treatment for anorexia.

## Introduction

This autoethnography approaches the diagnosis of anorexia as a social influence on a person. Recognising that diagnosis is a ‘powerful social tool, with unique features and impacts which deserve their own specific analysis’ (Jutel, 2009: 278), I examine my experience of ‘having’ the diagnosis in a treatment context.

Brinkmann (2016) describes the ‘being’ and ‘doing’ dimensions of a psychiatric diagnosis. ‘Being’ addresses psychiatric identity and the extent to which someone identifies with a diagnosis. The diagnosis of anorexia may be valued by some as a source of identity (Lavis, 2011; Rich, 2006; Warin, 2010). In her ethnography of people’s everyday experiences with anorexia, Warin (2010) found that the diagnosis was experienced by many as a desirable positioning and was something they worked towards. She describes how it can offer symbolic power, and that this arises from its mark of distinction and sign of belonging to an elite group. Participants in her study sought to mobilize the worth of the diagnostic label such that it became more to them than a medical diagnosis. Rather, it was ‘an empowering state of being, a friend, an enemy, and a way of life’ (2010: 7).

‘Doing’ refers to an action aspect of a diagnosis. Diagnoses are ‘something people do or perform relative to specific categories’ (Brinkmann, 2016: 31). Ringer and Holen (2016) suggest that in clinical settings, mental health patients learn to outwardly display their symptoms in recognisable ways in order to legitimise their need for care. Lavis (2011) addresses performative aspects of anorexia. She contends that bodily thinness visually defines what it means to be anorexic, thus highlighting the centrality of thinness to the performance of anorexia. She also argues that in treatment, patients learn what anorexia is and how it ‘should’ be done. Treatment replicates the practices that it assumes to be central to the condition, for example when ‘anorexic behaviours’ are pre-emptively responded to through rules which prevent patients from hiding or otherwise not consuming even tiny amounts of food. Through the clinic’s projection of anorexia, ‘many learn how to ‘do’ and ‘be’ the clinic’s anorexia’ (2011: 279).

Treatment for anorexia is associated with high rates of ‘drop-out’, relapse and chronicity (Hubert et al., 2013; Khalsa, 2017; National Institute for Health and Care Excellence [NICE], 2017). No treatment programme for adults leads to substantial levels of recovery or demonstrates sustained long-term benefits (NICE, 2017; Treasure and Cardi, 2017). Moreover, there are iatrogenic effects of inpatient treatment for anorexia. NICE guidelines state:

‘people with an eating disorder can become institutionalised by a long admission [. . .] a lack of change in their condition could indicate that inpatient treatment is harmful’ (NICE, 2017: 34).

Clinically-oriented studies have identified harm associated with punishing, rigid, coercive and/or confrontational treatment (Colton and Pistrang, 2004; Garner, 1985; Offord et al., 2006; Treasure et al., 2011). Narrative therapists have argued that treatment that seeks to control patients can inadvertently replicate anorexia itself (Epston and Maisel, 2013; Scott et al., 2013). Researchers have also suggested that a disproportionate focus on weight and food during treatment encourages individuals to continue their eating disordered behaviour by perpetuating concerns about food and weight (Eivors et al., 2003; Rance et al., 2017). Iatrogenic harm may also result from the comparing and ‘competing’ that can occur among patients, as individuals may learn new illness-related behaviours, seek to be ‘the best’ anorexic and become more unwell (Colton and Pistrang, 2004; Offord et al., 2006; Treasure et al., 2011; Warin, 2010).

This paper adds to these existing suggestions of iatrogenic harm. Through an examination of my own experiences, I suggest that being diagnosed and undergoing treatment ultimately intensified and prolonged my struggle with self-starvation. My analysis foregrounds the diagnostic category of anorexia, in terms of my relationship to it and the constructions of it I encountered.

## Methods

Autoethnographies ‘are highly personalised accounts that draw upon the experience of the author/researcher for the purposes of extending sociological understanding’ (Sparkes, 2000: 21). This autoethnography is a retrospective self-study which ‘combine(s) the power of the personal perspective with the value of analysis and theory’ (Wall, 2016: 8). I use memory, personal and medical documents, and existing literature to reconstruct, ‘tell’ and analyse my experiences with anorexia, diagnosis, and treatment in an adult Specialist Eating Disorder Unit (SEDU).

I underwent four long-term inpatient admissions (each between 4 and 8 months) in a SEDU within a 3.5 year period. The central goal of inpatient care is to reduce risk of physical illness and death through improved nutrition (NICE, 2017). In line with this, the main focus of my treatment was eating meals and achieving the target weight gain (0.5–1 kilo per week). Traditionally, inpatient treatment sought to restore weight to ‘normal’ (usually defined as a BMI of 20). However, ‘partial recovery’ programmes involving short inpatient stays and/or low body weight at discharge are now offered by most adult SEDUs (Goddard et al., 2013). This was the case in my treatment. A BMI of 20 was viewed as the ‘best’ outcome, but target weights were set individually and were sometimes lower. I had weekly individual therapy with a psychologist and attended regular mandatory group sessions (such as ‘body image’ and art therapy). There was not a singular, explicitly named psychological theory underpinning therapy on the unit, but much was oriented around cognitive behavioural therapy.

The personal documents informing the autoethnography are daily diaries that I kept during treatment. The medical documents include all clinical records relating to my admissions to the SEDU, and from my GP and community mental health team. I do not treat the documents as direct, unproblematic representations of events. Even when intended for personal use, diaries may be written with an imagined audience in mind (Riesssman, 2015) and medical records highlight only limited aspects of patients and their behaviour (Prior, 2003). However, their value is the insight they provide into how I made sense of daily life at that time (Berg, 2004).

There is great potential value in using one’s own life events as a source of sociological enquiry, especially when these events make deeply personal experiences unusually observable and provide access to data that is not otherwise available (Chang, 2008; Vryan, 2006). My autoethnography is orientated towards explicit analysis. It seeks to reach beyond my own experiences to address broader conceptual and theoretical issues (Anderson, 2006) relating to psychiatric diagnosis and identity.

This autoethnography is not intended as *the* truth of ‘what happened’. Rather, it is my sense-making of it. This is the value of autoethnography; it centralises my voice, avoids it being ‘othered’ by outsiders (Richards, 2008), and reflects the truth of my experience.

An important factor for evaluating autoethnography is its scholarly contribution (Chang, 2016; Le Roux, 2017). The main contributions of my autoethnography relate to the unintended consequences of being diagnosed with a psychiatric condition. Credibility and trustworthiness are also crucial to autoethnography (Chang, 2016; Le Roux, 2017). I have recalled events as honestly and accurately as possible. The use of unsolicited, primary documents that were produced by individuals who directly experienced my illness and treatment also enhanced credibility. However, the autoethnography is a public narrative, and as such I wrote it with an audience(s) in mind. This means that there are inevitably select, deeply personal aspects of my experiences that I have not shared. In addition, I omitted aspects of my experiences due to the impossibility of sharing *all* relevant experiences. Crucially however, I do not consider that this unavoidable ‘selectivity’ reduces the scholarly contribution.

Undertaking the autoethnography, relational ethics (Ellis, 2007) was a key concern. This refers to how, due to the researcher being identifiable, others are identifiable by association. I avoided this as far as possible by keeping others anonymous, restricting personal information about them, and omitting select details of my experiences. It has been suggested that such omissions can problematise the integrity, interpretation, and authenticity of the research (Delamont, 2009; Ellis et al., 2011). However, while I restricted information to protect others, this did not affect the key analytic insights I made.

Autoethnography can also harm the researcher themselves (Chatham-Carpenter, 2010; Dashper, 2015). When I began my autoethnography, I had been ‘recovered’ for approximately 7 years and was adept at coping with ‘triggers’. That said, being heavily immersed in my past was sometimes emotionally challenging. Importantly, difficulties were short-lived and without lasting impact.

## Autoethnography: Developing anorexia

It is difficult to define the beginning of anorexia. I could begin with the period in which I first recognised myself as ‘eating disordered’. Or I could begin with the time when ‘symptoms’ first began. I could reach further back still, to concerns about my body size in my early teens, or back yet again to my childhood where the roots of anorexia possibly lie. It is fair to say that there are many possible beginnings. Here, I have chosen one – my first decision to diet.

*At 16 years old I go into Topshop to try on a pair of jeans. As I stand in the changing room wearing them, I turn to look in the mirror and am struck by how fat I look. I cry. Later that day, after ruminating over the image in the mirror, it occurs to me that I will go on a diet and lose weight. In this moment of realisation, I sense a definite shift occur in my mind. I don’t take time to consider how or whether the dieting will work, I simply know that it is going to happen (memory recall).*

During the five or so years prior to my decision to diet I had increasingly considered that my peers were slimmer than me and that I was ‘too fat’. My upset in the changing room that day was an eruption of the sadness and shame about my body that had been building for some time. I dieted by restricting my intake of ‘fatty’ foods. Adopting the dominant ‘diet thinking’ of that time, I created my own set of rules to avoid fat, such as not consuming more than 30 g of fat per day. My dieting was ‘successful’; it wasn’t too arduous, I lost weight, and occasionally received positive comments from friends.

The diet lasted around a year before I reverted back to eating ‘normally’. About a year later, age 18, I returned to dieting, again motivated by a sense that I was ‘too fat’. However, this time I also started experiencing occasional episodes of overeating in which I would break my self-imposed diet rules and eat beyond fullness. Aged 19 I left the family home to move in with my close friend and go to university. My memories of this time – although mostly happy – are littered with episodes of starving and overeating. Strict dieting would lead to overwhelming hunger, and this in turn led to overeating, panic, further overeating, and attempts to fix the problem through stricter dieting.

### Being diagnosed

By age 20, the cycles of dieting and overeating had become extreme. I was in the third year of my sociology degree and focusing hard on assignments, with my sense of personal worth becoming increasingly attached to academic success. Retrospectively, I view my binge eating as an understandable physiological response to my restrictive eating, but at that time, I made no link between the two extremes of behaviour. Bingeing felt like a personal failing and motivated to stop, I sought help from a charity-run eating disorder service (EDS). I did not consider myself to have a ‘serious’ eating disorder but suspected that I might have a ‘mild’ form of bulimia (minus the vomiting). I met with a counsellor at the charity and explained my eating habits. She suggested that perhaps it was not bulimia I was experiencing, but anorexia. I was surprised. I knew that my dieting between binges was extreme and that I was slightly underweight, but I had not thought of it as anorexia. Surely I was not thin enough, and surely I liked food too much?

Thinking of myself as ‘anorexic’ felt like a presumptuous overstatement of my weight loss. Further, maintaining food restriction was like walking a tight rope; at any moment I could slip, lose control of my eating and gain weight. Associating myself with the diagnosis invoked anxiety that I was not good enough to warrant it (Lavis, 2011). Yet, I also *wanted* to identify with anorexia. The word felt rewarding, marking out a level of seriousness I had never thought I would achieve. I liked the idea that I had taken food restriction far enough to be considered clinically ill.

After the diagnosis had been suggested, I did not continue with appointments. During the summer after completing my degree I began binge eating and it became too painful to confront my eating patterns, so I no longer wanted help. By around 9 months later (age 21) my eating had settled into a relatively ‘normal’ pattern. While studying for a masters degree and living in a rented flat with my then partner, I started work as a research assistant at the university where I was studying and shortly afterwards developed an abscess on my gums. The abscess made eating difficult and I consequently lost a little weight. This pleased me and motivated a further period of food restriction which led to intentional, sustained and marked weight loss. One evening I had been unable to finish eating a slice of pizza because of the abscess. Three to four months later, I was meticulously monitoring and severely restricting my calorie intake. I was thrilled by the discovery that I was able to lose significant amounts of weight and felt intoxicated by my new extremes of food refusal.

However, over time, my restricted eating shifted from being something that I simply wanted to do, to something that was also extremely difficult. I felt increasingly weak, everyday tasks became arduous and I struggled to focus on work. Recognising the extent to which I felt ‘stuck’ in restrictive eating, I came to perceive myself as someone with anorexia. I had first begun to understand myself in terms of the diagnosis when it was suggested by the counsellor and this understanding had strengthened as my weight loss continued and I read more about anorexia. Four months into this bout of weight loss I returned to the EDS and my GP and described myself as having anorexia. At this point, a diagnosis of anorexia was officially recorded.

Over the following 3–4 months, I continued with weekly appointments at the EDS and my GP and expressed a desire to change due to how much I was suffering. I lacked energy and felt light-headed and constantly cold. My life was very limited and monotonous, my mood persistently low and I never laughed anymore. Yet, I simultaneously did not want to stop restricting food. I was captivated by weight loss and unable to imagine living without this exciting ‘thing’ in my life. I relished the secrecy and privacy; I had something that was mine and that no one else could touch. The private knowledge that I was successfully starving felt like a reassuring, comforting presence and nothing else really mattered, so long as I was losing weight. Torn between staying in my pseudo-protective state of starvation, and an increasing sense of guilt for the ‘proper life’ I was not living, I was highly ambivalent.

At the EDS I was given guidance on increasing my food intake, which I intended to follow, but did not.

I haven’t tried to lose more weight, but I consciously let it happen. I want to get better but [I feel] relief at seeing the scales go down [. . .] I can literally feel the anxiety wash away when I count up a days calories and realise I’ve ‘beaten’ my ‘target’ (Diary, 22nd July 2006).

About 3 months after the diagnosis had been recorded, the GP asked me whether I would consider an admission at a SEDU. Wanting to change but feeling unable to do so, I agreed. In the 6-week wait to be admitted, I became increasingly desperate.

*I am at home in my flat having a shower. I have the shower water very hot in an attempt to warm me up. This is futile. The cold, seemingly impossibly, continues to radiate from the inside, even while the scorching hot water creates red marks all over my skin. I begin to feel sick and faint and decide to climb out of the shower. I am weak and breathless with a constant buzzing ringing through my ears. As I bend down while drying myself, the veins in my arms bulge from my skin as if they might explode. I feel unreal. I stand on the scales and the red digits flash a new number; I have lost another pound. In that moment, I don’t feel the euphoric elation I usually do when losing weight. Instead, I think: ‘What am I doing? I really should eat.’ It occurs to me that I cannot. I go to my bedroom, sit on the bed, and cry (Memory recall).*

Despite my desperation, I questioned the legitimacy of my anorexia. Making reference to a lay construction of the diagnosis, I considered I fell short of ‘proper anorexia’. I imagined this involved being emaciated to a greater extent than I perceived myself to be, and actively fearing food. Not only did I not fear food, but I desperately wanted to eat it, I just could not allow myself to for worry about weight gain. I also imagined that proper anorexia occurred for people who were so deeply troubled that they lacked appetite, whereas I was trying to squash a desperate appetite. There therefore seemed to be something pretend about what I was doing, and as though my anorexia was not serious enough for inpatient treatment.

### Encountering constructions of anorexia in treatment

I arrived at the unit on a Monday morning, and a few hours later ate my first meal. It was just me and one staff member. I still remember the saltiness of the cottage pie as I ate slow and steady mouthfuls and the small pools of butter sitting around the boiled potatoes. In hindsight, I wonder how I simply sat down and finished it having been unable to eat more while at home. I suppose I was so bound up in the expectation that this is what I would do (completing meals was non-negotiable) that it did not occur to me that I might not.

During the first few weeks I got used to the hospital routines and what was expected of patients. In some respect I enjoyed eating and was grateful that I ‘had to’, but I worried that this proved the fraudulence of my anorexia. Aware that a patient in a SEDU should be *not* wanting to eat, as appeared to be the case for other patients, I kept these concerns to myself.

Being forced to eat a (half-sized) normal meal was almost a welcome relief [. . .] A proper anorexic would no way be pleased at the sight of the plate of food I just ate (Diary, 9th October 2006).

My diaries from this time allude to the two subjective positions that I shifted between; one was that my anorexia was pretend (I was not anorexic *at all*), and the other was that I was not a ‘good’ anorexic (I was not anorexic *enough*).

The fact that I’m not trying to get out of eating butter when I eat a meal here means I’m not trying hard enough. I was never a good anorexic in the first place because surely then the butter would be harder? I’m a fraud case, I’m not anorexic (Diary, 14th October 2006).

These positions were heightened by comparisons with other patients, who concerned me immediately upon being admitted.

All the other girls have been loads skinnier than me. Some of them have been in [general] hospital because they’ve been that underweight. And then there’s me (Diary, 10th October 2006).

My sense of inadequacy was also enhanced by well-meaning comments from staff. They told me I had a good chance of recovery because this was my first phase of severe anorexia and I had voluntarily sought help. I took this to mean I was not as anorexic as other anorexics.

Initially, the weight gain did not feel too difficult. I did not want to lose my thinness, but something about the change in context (hospital rather than home) made the scale reading relatively meaningless. However, a few months in, I could see the changes on my body and things became hard:

Struggling today with my size. I feel so much bigger these last few days [. . .] There have been two new admissions [. . .] It really screws me up seeing extremely thin people [. . .] they are pleased they are not as fat as I am (Diary, 18th January 2007).

Despite these struggles, I continued treatment. Twenty-four weeks in, I temporarily reached a BMI of 19 before undercutting my meal plan. As I neared the end of my admission, my weight was becoming my own responsibility again (I was now eating away from staff), and I struggled with permitting myself to eat. In addition, I dreaded my imminent discharge.

Being an inpatient for 8 months had disconnected me from ‘the real world’. I had previously wanted to recover for the sake of my personal relationships and job, but over time these lost their motivational influence. Rather than being immersed in ‘normal life’, I had been in a unit where anorexia was ever present – in other patients, in the treatment programme, in my daily interactions. I was used to anorexia being the standpoint from which I related to others, and the way that I understood myself. I had also been exposed to a new, ‘other’ anorexia, which was more than my own self-starving. It was an anorexia that was bound up with clinical activity and involved being *really sick* – multiple hospital admissions; concerned doctors; deathly low weights; being detained; physical complications; bed rest; threats of tube feeding.^1^

I saw this construction of ‘serious anorexia’ in others and heard it in their treatment histories. I saw it in the ‘high dependency’ rooms on the unit, with special mattresses for the *other* patients whose weight was so low they would otherwise get bed sores. It was detailed to me by staff, who told of the threats anorexia posed to me in the future if I did not ‘recover’ now. The SEDU privileged this ‘serious anorexia’, which for me carried intangible overtones of success and achievement. As Lavis (2011) argues, the clinical practices associated with anorexia form part of the desire for it: ‘through both its legitimisation of, and intervention into, ‘anorexia-as-illness’, the clinic itself [. . .] is central to pro-anorexic desire’ (p. 279). To me, it seemed remarkable, and was elusively appealing. Beginning with this first admission, gradually (and imperceptibly at that time), my ‘wanting’ was shaped by the SEDU and clinical discourse I was immersed in. My sense that I needed to lose weight was no longer *only* about wishing to return to the ‘psychological benefits’ of food restriction. It was also about desiring the diagnosis of anorexia itself (Lavis, 2011; Warin, 2010), and the construction of it I had encountered in treatment.

Darmon (2017) contends that when someone diagnosed with anorexia enters a hospital institution (such as a SEDU), the institution seeks to reverse the individual’s commitment to anorexia by redefining their behaviours and intentions as pathological and replacing them with ‘healthy’ ones. As the individual gets ‘on board’ with this process and internalises the hospitals perspective, they actively invest in ‘recovery’. Recognising oneself as ‘anorexic’ is a necessary step in this process. For me, this recognition occurred prior to entering treatment. However, being in treatment strengthened this self-view by increasing the scope of my experiences that were defined as pathological. Even previously ‘normal’ experiences were retrospectively understood as ‘anorexic’. In my first admission I was asked to write a weight and body history. I wrote about how, age 7 in gymnastics class, I noticed the curve of my belly in my leotard; how I compared my figure to those of my friends at secondary school; how I had started dieting at age 16. Rather than being typical childhood experiences, they were now viewed through the lens of anorexia as precursors to disorder.

## Repeated admissions: Pursuing anorexia

Once discharged, I continued the weight loss that had begun on the unit, although I was not really sure why. I returned to work as a research assistant after 2 weeks, but felt incapable of doing my job and was extremely lonely. An unshakable feeling of displacement cemented my continuing weight loss, and I began to more actively seek a return to anorexia, now with a clear picture in mind as to how this should ‘look’. I restricted my diet much faster than I had previously, and as a result felt far worse. I had constant nausea and a low-pitched hum ringing through my head, which I imagined to be a physical manifestation of my low mood. My community team’s concerns for my physical health escalated when a blood test indicated muscle wastage and 8 weeks after discharge from the unit, they made a referral for me to return.

The first few days of my second admission I was utterly disoriented and as I began eating, overwhelmed by the sensation of food in my stomach. The culture on the unit was different from how it had been previously, due to there being some patients who were under section and (more or less openly) not ‘complying’. Their presence underlined to me the possibility of being on the SEDU as an involuntary patient. I found the idea that, in contrast to them, I was *choosing* to be in treatment distressing:

Lauren spoke about feeling undeserving and confused about why she’s here i.e. feels ‘normal’ and ‘too big’ etc [. . .] expressing much distress at ‘choosing’ to be here, much guilt after and during eating (medical notes, SEDU, 13th July 2007).

In response to this distress, on the third day, I did not finish my breakfast. By the next day, I had almost entirely stopped eating. I was told that I would be sectioned if I did not eat my lunch, but I could not wholly believe this would happen (I was not a serious-enough anorexic). This ultimatum defined the ‘anorexic’ option for me, and I now felt I ‘had’ to *not* eat, otherwise I would be taking the less-anorexic route. So I did not even try. Medical notes from this time describe:

Lauren lacks the ability to ‘hear’ how ill she is [. . .] Her reasoning is illogical, she refuses to let herself be encouraged to be fed [. . .] (she) is currently physically unstable and lacks the insight to agree informally to treatment (Medical notes, SEDU, 16th July 2008).

That afternoon I was placed on bedrest and constant observations^2^ and sectioned^3^, on the grounds of an abnormal electrocardiogram (ECG) reading, my BMI dropping below a certain critical level and staff’s resulting wish to be able to tube feed me if I did not eat.

I stayed on bedrest for a week. During this time, I was taken to the general hospital site for a heart echo and scan of my lungs, due to health concerns including a suspected blood clot on my lungs. I recall my psychiatrist stood talking to me while I was on bedrest and saying starkly ‘Lauren, your anorexia is killing you’. In that moment, I wanted so badly to connect to what he had said, to find something to cling to amidst the precariousness of the vision I had of myself as properly anorexic. Indeed, serious medical conditions, in playing the fine line between life and death, can give more credibility to the diagnosis of anorexia (Warin, 2010). But the words were ghost-like and ungraspable, unable to do the job of confirming my anorexia.

The results of the heart echo and scan were ‘clear’, but due to ongoing concern for my health, staff decided to tube feed me. When I was told, I was overcome with distress and sedated. The idea of gaining weight was difficult enough, but, following such an intense period of starvation, the thought of not even having the consolation benefit of eating food was unbearable (although believing that an anorexic should not be wanting to eat, I could not have said this). I also felt overwhelmed by the idea of liquid calories entering into my body without my actions (eating) mediating the process.

Due in part to my persuasive negotiations with staff, I was not fed through the tube for long, although it stayed in situ for a few weeks so that it could be used if I refused food. Despite me not wanting to be tube fed, wearing the tube allowed me to negotiate my positioning, both personally and socially, as someone who was eating but none the less ‘good at anorexia’ (Lavis, 2011). I could attribute my eating to the threat of being tube fed, positioning me as someone who ‘had no choice’, and locating the responsibility for my eating elsewhere (Lavis, 2011). The tube functioned as a ‘personal and public signifier of [my] anorexia’ and crucially enmeshed with my identity (Halse et al., 2005: 11).

In addition, the tube signified to me a particular patient ‘type’; the ‘bad anorexic patient’. ‘Bad patient’ is a construction of anorexia that overlaps with ‘serious anorexia’ and denotes someone who is defiant, non-compliant and refuses food at all costs. It is reflected in clinical discourse. In hospital-settings, those diagnosed with anorexia are generally assumed to be ‘non-compliant’ and ‘difficult’ (Darmon, 2017) and are ‘notorious for their often concerted resistance to therapeutic processes’ (Gremillion, 2003: 3). Literature describes how ‘anorexics’ are ‘difficult to treat’ due to them being devious (Bruch, 1988) and actively subverting therapy (Vitousek et al., 1998). The construction was evident to me in the SEDU. I lived alongside other patients who were publicly known to be ‘non-compliant’. The construction was reflected in the preventative rules on the SEDU. These rules, such as having to be observed post-meal and not being allowed free access to water, were designed to prevent ‘deceitful’ behaviours such as water loading,^4^ hiding food and vomiting after meals. Often, it was the very existence of these rules that made me aware of the behaviours. Discussing water loading, Gremillion (2003) notes that treatment environments ‘underline this as a possibility for patients by institutionalising a response to it’ (p. 14). Crucially, I experienced these possibilities as expectations. When I was exposed to the suggestion of such behaviours but was not engaging in them, I found my anorexia wanting (Lavis, 2011). The SEDU assumed how individuals with anorexia behave (deceitfully) and embedded this assumption in its treatment responses. These assumptions informed my understanding of how anorexia should be done. There were numerous ‘secretive’ behaviours I engaged in, such as exercising, hiding food, and water loading. These were intended to maintain my anorexia by resisting weight gain, but they also allowed me to reassert my self-determination and retain my anorexic identity (Rich, 2006). I felt empowered by the knowledge that I was acting in these ‘extreme’ ways. Witnessing myself acting how a ‘serious’ and ‘non-compliant’ anorexic acts felt affirming.

About 8 weeks into the second admission, I was placed on bedrest to control the secretive exercise I had been doing. The initial weight change during bedrest was unbearable, more so than at any previous time:

The inevitable happened, I gained weight and I can’t cope [. . .] I can’t even write about it yet because I can’t accept it [. . .] I feel like I’m cracking up (Diary four, 18th September 2007).

Weight gain was intensely difficult due to my awareness that I was losing the thinness that made me feel psychologically safe and the sensation of becoming larger was intolerable. But more than this, weight gain was hard because it meant treatment was ‘taking anorexia away’. I was ‘cracking up’ because it was symbolic of my undoing (Lavis, 2011) and undermined my belonging to anorexia. Fox and Diab (2015) suggest that it is superficial to reduce the distress of ‘refeeding’ to a symptomatic ‘fear of gaining weight’. Rather, it results from weight gain challenging the way that one perceives oneself and undermining one’s ‘anorexic identity’. It also invalidated my suffering. In the SEDU, ‘more thin’ was equated with ‘more anorexic’ (Lavis, 2011), and a higher BMI positioned me as less deserving of care. I wrote in my diary ‘[When my] BMI is higher I won’t be able to struggle as much’ (Diary, 25th August 2007).

On bedrest, there was nothing I could do to prevent weight gain so I stopped fighting it. Over the 2–3 months that followed, I begrudgingly accepted my weight increasing. In the main, I complied with treatment. This allowed me to negotiate a BMI target of 16, by persuading staff that I would maintain at this level. My section was lifted towards the end of the admission. Unable to shake the guilt of voluntarily eating, I self-discharged and began losing weight. Two further admissions followed, both of which involved me being detained.

I have descried how, beginning with my first admission and then progressing over time, I came to value an identity based on serious anorexia and being a bad patient and was self-conscious of how I performed it. However, as my admissions progressed, these positionings also became problematic.

## Hospital restrictions and a pathologised identity

During my last two admissions, my struggle against the treatment programme that had occurred previously continued. Moreover, staff increasingly saw me as ‘risky’, due to being ‘medically unstable’ and having low mood. This is reflected in my clinical notes, which describe me as having ‘significant compromised health’ and as a ‘massive risk to herself’, as well as being at risk of suicide.

During these admissions especially, my inner world had become bleak and chaotic. My treatment reflected this, as it became ever more restrictive. The more I adopted the ‘anorexic role’, and the more time I spent under restrictive treatment conditions, the more I was distanced from ‘normal life’. In turn, I gripped more tightly onto anorexia. Lavis (2011) describes how a recognition of the damage that anorexia has done to one’s life can increase pro-anorexic desire. I had lost all motivation to pursue a career, had no home, no partner, and was accustomed to living in an institution. In these conditions, seeking to do anorexia well (instead of normal life) made sense. Seed et al. (2016) note that the loss of friendships, normal life and life skills that results from extended periods of inpatient treatment and repeated detention under the Mental Health Act can contribute to dependence on the treatment environment. This in turn makes it harder to ‘let go’ of one’s ‘anorexic self’. I certainly felt this spiralling impact.

I spent most of the last two admissions feeling ‘stuck’. I wanted anorexia, and as alternative options seemed to narrow off, I became unable to imagine life without it. Change did not seem possible. However, during the last 3 weeks of my final admission, my perspective began to gradually shift, largely due to a wish to avoid future hospital admissions. I was now finding the lack of autonomy that came with being in treatment utterly intolerable.

I felt frustrated when staff made pre-emptive decisions due to my perceived ‘risk’. Taking an example from my last admission, I had submitted to staff numerous requests prior to a meeting, such as being allowed home for leave. At this point, there was an expectation that I would be discharged 11 days later, so I argued that leave would be helpful preparation for going home. During the meeting staff decided that my section would be lifted but that my requests would not be met:

She is not close to recovery [and] is very impulsive [. . .] this unit is not the right place for Lauren, as she is eating, but not doing the psychological work [. . .] take off section today, no leave (Medical notes, SEDU, 6th January 2009).

To staff, I was behaviourally complying by eating, but not taking on the unit’s definitions of progress and recovery (Darmon, 2017). Feeling unfairly treated, I declared that I would go on leave anyway now that my section was lifted. In response, staff reversed their decision about the section. I then became even more upset, in turn increasing how ‘risky’ I was judged to be. Staff reacted by changing my cigarette breaks from ‘unescorted’ to ‘escorted’, and my upset deepened further. Staff notes describe me as being unable to channel my anger appropriately. I recall well how asphyxiating and disabling this series of moves felt.

I also frequently felt ‘unheard’ and my reasoning invalidated due to my inability to escape an anorexic framing. False accusations of ‘non-compliance’ felt impossible to refute, and this can be understood in terms of Goffman’s (1961) ‘looping’. Here, my reactions to my situation were collapsed back into the situation itself; my denial of ‘anorexic behaviour’ was interpreted as symptomatic of anorexia and I was unable to defend myself in the usual way. A memorable example is from my final discharge meeting. Just prior to this, I had requested a change in care coordinator citing the reason of a ‘personality clash’ having felt unable to develop rapport with her. At the start of the meeting, the psychiatrist questioned my ‘true’ intentions and accused me of wanting a new care-coordinator because ‘the anorexia’ wanted someone new to manipulate. Finding a way of believably rejecting this suggestion was impossible.

In treatment, anorexia was reductively constructed as a pathologised, medicalised condition, and while in some ways affirming, this also sometimes led me to feel misunderstood, invalidated and stereotyped (Eli, 2014; Boughtwood and Halse, 2010; Malson, 2004; Rich, 2006). Anorexia became the overriding source of my identification, leading to my behaviour being automatically interpreted as symptomatic of illness, resulting in me feeling powerless (Boughtwood and Halse, 2010; Eli, 2014; Malson, 2004; Smith et al., 2014; Seed et al., 2016). In addition, being unable to make my own decisions felt child-like and I found this humiliating.

Eventually, I realised that the longer I was in and out of treatment, the further the walls would close in. Wanting something different, I tentatively opened up in my mind to the idea of letting go of anorexia. I was discharged for the final time with a strict plan dictating immediate admission to a psychiatric unit or general hospital should my weight drop below an agreed range. I initially maintained my weight, but after around 3 months I began to binge eat uncontrollably. The years that followed involved a painful and turbulent struggle to come to terms with a rapidly changing body and identity as I was no longer classified as ‘anorexic’.

## Conclusions: Being and doing anorexia

I have addressed the ‘being’ and ‘doing’ (Brinkmann, 2016) of my anorexia diagnosis. I have shown how, over time, I came to value an anorexic identity, and that this had implications for how my suffering was ‘done’. The diagnosis of anorexia was a positioning I strove towards and which sometimes felt empowering (Lavis, 2011; Warin, 2010). While this striving was fractured by occasions of feeling disempowered and silenced, on the whole I sought to inhabit my diagnosis. I did this via a self-conscious monitoring of whether I was doing anorexia ‘properly’. Through the constructions of anorexia that were detailed to me in treatment, I learned new ways of doing anorexia, living up to the SEDUs implicit expectations of how ‘an anorexic’ behaves and how anorexia manifests psychologically (Lavis, 2011). Clinical signifiers of anorexia, such as tube feeding and a low BMI acted as identity markers, proving my anorexia and warranting it a ‘serious’ status (Halse et al., 2005; Seed et al., 2016) . Yet, any sense of having done anorexia ‘well’ was only ever fleeting and fragile. This made leaving anorexia all the more difficult, because there was a sense that if I did it again, one more time, I might just achieve it. These diagnostic-dynamics, combined with other effects of long-term inpatient treatment such as detachment from ‘normal life’, had an ultimately detrimental impact on my condition.

This article has illuminated hidden consequences of being diagnosed with anorexia and undergoing inpatient treatment, giving insight into iatrogenic aspects of these. I have suggested that being diagnosed and treated prolonged my experience of self-starvation. However, I will never know what the trajectory of my self-starvation would have been had I not received the clinical intervention I did, nor whether or how it would have evolved had the diagnostic concept of ‘eating disorders’ not existed. It is also vital that I recognise that the physical clinical interventions I received likely saved my life on occasion. Jutel (2011) argues that ‘Diagnoses are social categories that organise, direct, explain and sometimes control our experiences of health and illness’ (p. 145). I believe that in my case, the diagnosis – and all the ways it was communicated to me in treatment – served to channel my self-starvation into a recognisable and sustained ‘anorexia’.

## Notes

1. ‘Tube feeding’ refers to the use of a nasogastric feeding tube. In treatment for anorexia, it is used as an alternative or addition to the patient eating food, to ensuring they gain weight.

2. ‘Constant observations’ refers to the mental health nursing practice of maintaining high levels of observations of patients for their safety.

3. I was placed on a section 3 which is used to detain someone for treatment 6 months.

4. Water loading refers to the practice of distorting one’s weight so that it appears higher by consuming large volumes of liquid.
